# Vitamin K shot in newborn babies: An unprecedented sequelae

**DOI:** 10.1016/j.amsu.2022.103942

**Published:** 2022-06-04

**Authors:** Muhammad Ayyan, Afra Zahid, Muhammad Ehsan, Qasim Mehmood, Irfan Ullah, Muhammad Sohaib Asghar

**Affiliations:** Department of Internal Medicine, King Edward Medical University, Lahore, Pakistan; Kabir Medical College, Gandhara University, Peshawar, Pakistan; Department of Internal Medicine, Dow University Hospital-Ojha Campus, Karachi, Pakistan

**Keywords:** Vitamin K, Pediatrics, Infants, Bleeding, Deficiency

## Correspondence

Vitamin K, a fat-soluble vitamin, is an essential nutrient for the human body whose major role is to assist in the production of several coagulation factors. Unlike many other vitamins, vitamin K is not usually used as a dietary supplement [1]. However, all newborns have very low levels of vitamin K in their blood. This is attributed to the impermeability of the placenta to vitamin K [2] and a very low amount of the vitamin in breast milk (1 μg per liter), irrespective of how many supplements a woman takes [3]. The low levels of vitamin K in infants make them susceptible to a potentially life-threatening condition called vitamin K deficiency bleeding (VKDB), which can occur in all infants up to the age of 6 months if they do not receive a vitamin K shot. There is a high mortality rate of 20% associated with late vitamin K deficiency bleeding [2]. The affected infants can bleed into their brain or gut, which is likely to go undiagnosed and this leads to high mortality and long-term adverse effects associated with the condition. Bleeding into the brain can cause permanent neurological damage [2] and rarely, some severe long-term adverse effects including gross motor skills deficits, cognitive or developmental anomalies, and organ failure [3].

Vitamin K deficiency bleeding (VKDB) can be divided into three types: early, classic and late. Early vitamin K deficiency bleeding occurs within the first 24 hours after birth. In most cases, the vitamin K deficiency bleeding is secondary, implying that the newborn has an underlying bleeding disorder which is exacerbated by vitamin K deficiency, or the baby was born to a mother who was taking drugs that inhibit vitamin K, such as antiepileptic drugs, blood thinners like warfarin, and some antibiotics. Classic vitamin K deficiency bleeding occurs within the first week after birth. Late vitamin K deficiency bleeding occurs in babies 2–24 weeks of age. It is a severe condition with high mortality i.e., 1 out of 5 babies with late vitamin k deficiency bleeding die, and out of every 5 survivors, 2 suffer from long-term neurological damage. All three forms of vitamin K deficiency bleeding can lead to bleeding in the gut or the brain [3].

To prevent this disastrous disease, a single Vitamin K shot is given which can almost entirely reduce the risk of vitamin K deficiency bleeding. A 0.5 mg or 1 mg (varies with birth weight) vitamin K shot is given intramuscularly to all babies at birth. All the other ingredients in the shot are safe and are added either to increase the absorption of vitamin K or to maintain the pH and moisture in the shot [3]. Some parents prefer oral vitamin K administration to the intramuscular shot. The Canadian Paediatric Society (CPS) has recommended that a 2.0 mg dose of oral vitamin K be given to newborns, first within 6 hours of birth, then at 2–4 weeks, and then, at 6–8 weeks of age [4]. Oral Vitamin K is said to be less effective than IM shot [3] because of the better absorption through the intramuscular route but according to a review written by Julien [5], the oral and IM routes of vitamin K administration are comparable in terms of efficacy if the oral doses are duly completed. However, the oral route is not suited for newborns with biliary atresia [5].

A qualitative study conducted in 2019 outlined the reasons for refusal of the vitamin K shot by the parents for their newborns [6]. According to its findings, some parents thought that the risks outweighed the benefits and were especially concerned about the other ingredients added to the shot. Another reason for refusal was the lack of trust in the healthcare workers and the pharmaceutical companies, whom the parents believed to be working in their own interest rather than the interest of the newborn. Some parents refused to get the shot for their newborn based on their experiences with their previous children, for whom they were not advised or offered vitamin K shot, while others wanted to adopt the ‘natural’ approach for their children, and were of the view that the vitamin K deficiency, being natural, was normal and did not need to be corrected [6].

There is non-uniformity when it comes to the practice of vitamin K administration [7]. This can be attributed to the ambiguous global policy due to the two different recommendations given by WHO. According to the first recommendation, “All newborns should receive a 1.0 mg IM injection of vitamin K at birth.” [8] The second recommendation states that “Neonates requiring surgical procedures, those with birth trauma, preterm newborns, and those exposed in utero to maternal medication known to interfere with vitamin K are at especially high risk of bleeding and must be given vitamin K” [8]. Due to these recommendations, many people believe that it is not necessary to administer the vitamin K shot to all newborns and should only be given to those with certain risk factors. However, the updated Pocket Book of Hospital care for Children by WHO recommends the administration of vitamin K to all newborns [9]. Some other barriers to the use of vitamin K include restricted access, supply chain logistics, the attitude of the suppliers, and the restrictions imposed by the suppliers [7].

According to a systematic review, the low-income areas showed higher rates of vitamin K deficiency bleeding. This difference remained even after the introduction of vitamin K administration and could be attributed to the differences in ethnicity, seasonality, and geolocation (higher incidence in summer and in the tropics) [10]. A case series reported that the rural areas of East Malaysia showed a higher incidence of such cases due to the increased frequency of unregistered home births [11]. People opt for home deliveries in low-income areas due to the cost of transport and other difficulties associated with reaching the hospital on time. This causes an increased number of unregistered births that do not receive the vitamin K prophylaxis, leading to a greater risk of vitamin K deficiency bleeding in those regions. Exclusive breastfeeding and lack of awareness among the people are other important reasons for such cases in these areas.

The developing and low-income countries have been facing an alarming number of avoidable neonatal deaths due to vitamin K deficiency bleeding. The policymakers and Health Sector need to focus on this issue and improve the access and availability of the prophylactic vitamin K shot following the recommendations of WHO, while ensuring the strict implementation of all the policies made in this regard. The parents of newborns should be counseled properly and the importance of the shot emphasized with evidence in order to decrease the number of cases of vitamin K deficiency bleeding.

## Ethics statement

No ethical requirements were reported for this study.

## Funding

None.

## Author contributions

M.A, A.Z, and M.E conceived the idea; M.A, A.Z, M.E, and Q.M collected the data; I.U and M.S.A analyzed and interpreted the data; M.A, A.Z, M.E, Q.M, and I.U did write up of the manuscript; and finally M.S.A, I.U, and Q.M reviewed the manuscript for intellectual content critically. All authors approved the final version of the manuscript.

## Provenance and peer review

Externally peer reviewed, not commissioned.

## Declaration of competing interest

The authors declare that there is no conflict of Interest.
